# Poly[[{μ_4_-1,2-bis­[(3-cyano­benzyl­idene)hydrazono]-1,2-diphenyl­ethane}bis­(trifluoro­methane­sulfonato)disilver(I)] benzene solvate]

**DOI:** 10.1107/S1600536808012853

**Published:** 2008-05-07

**Authors:** Hong Liang Li

**Affiliations:** a Department of Chemistry, Dezhou University, Dezhou Shandong 253023, People’s Republic of China

## Abstract

In the title complex, {[Ag_2_(CF_3_O_3_S)_2_(C_30_H_20_N_6_)]·C_6_H_6_}_*n*_, the two independent Ag^I^ ions are each coordinated by two N atoms and one O atom in a ‘T-shaped’ geometry. In the crystal structure, 1,2-bis­[(3-cyano­benzyl­idene)hydrazono]-1,2-diphenyl­ethane ligands act as bridging ligands and each coordinates to four Ag^I^ ions, resulting in a one-dimensional chain structure. The crystal structure is stabilized by weak inter­molecular C—H⋯O hydrogen bonds.

## Related literature

For a related structure, see: Liu (2008 ▶).
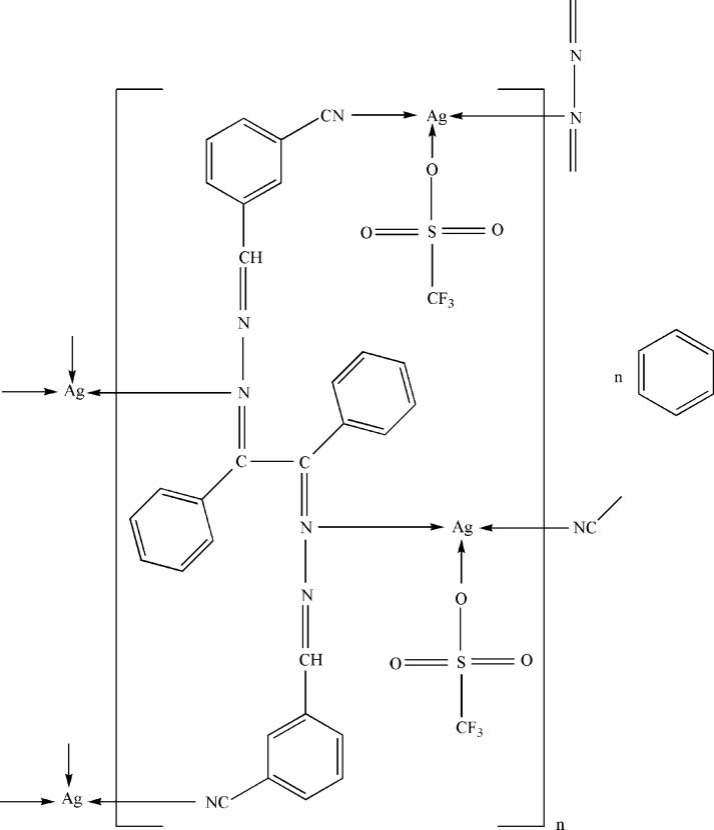


## Experimental

### 

#### Crystal data

[Ag_2_(CF_3_O_3_S)_2_(C_30_H_20_N_6_)]·C_6_H_6_
*M* *_r_* = 1056.51
Monoclinic, 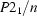

*a* = 10.8308 (9) Å
*b* = 22.0864 (18) Å
*c* = 17.0949 (13) Å
β = 90.420 (2)°
*V* = 4089.2 (6) Å^3^
*Z* = 4
Mo *K*α radiation
μ = 1.14 mm^−1^
*T* = 298 (2) K
0.33 × 0.23 × 0.12 mm

#### Data collection

Bruker SMART APEX CCD diffractometer
Absorption correction: multi-scan (*SADABS*; Sheldrick, 1996 ▶) *T*_min_ = 0.705, *T*_max_ = 0.875
23919 measured reflections
8891 independent reflections
6637 reflections with *I* > 2σ(*I*)
*R*_int_ = 0.026

#### Refinement

*R*[*F* ^2^ > 2σ(*F* ^2^)] = 0.048
*wR*(*F* ^2^) = 0.134
*S* = 1.04
8891 reflections
541 parameters
H-atom parameters constrained
Δρ_max_ = 1.16 e Å^−3^
Δρ_min_ = −0.29 e Å^−3^

### 

Data collection: *SMART* (Bruker, 1997 ▶); cell refinement: *SAINT* (Bruker, 1997 ▶); data reduction: *SAINT*; program(s) used to solve structure: *SHELXTL* (Sheldrick, 2008 ▶); program(s) used to refine structure: *SHELXTL*; molecular graphics: *SHELXTL*; software used to prepare material for publication: *SHELXTL*.

## Supplementary Material

Crystal structure: contains datablocks I, global. DOI: 10.1107/S1600536808012853/lh2619sup1.cif

Structure factors: contains datablocks I. DOI: 10.1107/S1600536808012853/lh2619Isup2.hkl

Additional supplementary materials:  crystallographic information; 3D view; checkCIF report

## Figures and Tables

**Table:** 

Ag1—N4^i^	2.196 (3)
Ag1—N5	2.249 (3)
Ag1—O1	2.568 (3)
Ag2—N3^ii^	2.169 (3)
Ag2—N1	2.217 (3)
Ag2—O4	2.592 (3)

**Table:** 

N4^i^—Ag1—N5	165.35 (12)
N4^i^—Ag1—O1	85.26 (13)
N5—Ag1—O1	108.59 (11)
N3^ii^—Ag2—N1	157.73 (13)
N3^ii^—Ag2—O4	94.85 (11)
N1—Ag2—O4	103.97 (10)

Symmetry codes: (i) 
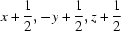
; (ii) 
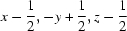
.

**Table 2:** Hydrogen-bond geometry (Å, °)

*D*—H⋯*A*	*D*—H	H⋯*A*	*D*⋯*A*	*D*—H⋯*A*
C33—H33⋯O6^i^	0.93	2.57	3.486 (5)	168
C26—H26⋯O3^iii^	0.93	2.52	3.406 (5)	159
C22—H22⋯O5^i^	0.93	2.56	3.382 (4)	148
C6—H6⋯O5^iv^	0.93	2.47	3.178 (4)	133

Symmetry codes: (i) 
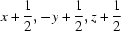
; (iii) 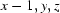; (iv) 
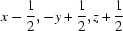
.

**Fig. 1.**
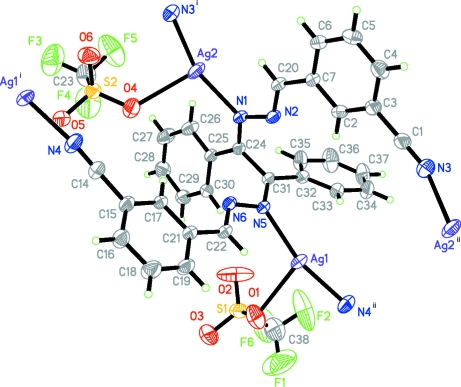
Part of the one-dimensional chain of the title structure showing the coordination of the two independent AgI ions. Displacement ellipsoids are shown at the 30% probability level and the benzene solvent has been omitted for clarity. [Symmetry codes: (i) x-1/2, -y+1/2, z-1/2; (ii) x+1/2, -y+1/2, z+1/2.]

**Fig. 2.**
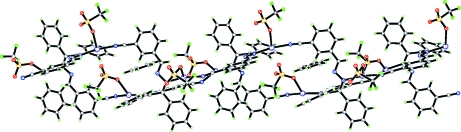
Part of the one-dimensional chain structure showing the formation of a 24 atom ring.

**Fig. 3.**
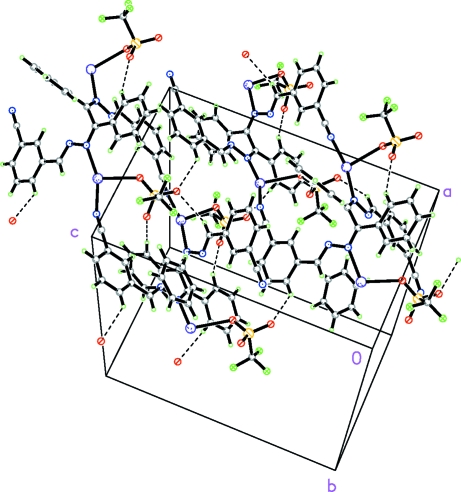
Unit cell and part of the crystal structure showing weak C—H···O hydrogen bonds as dashed lines.

## supplementary crystallographic information

### Comment

1,2-Bis[(3-cyanobenzylidene)hydrazono]-1,2-diphenylethane is a useful multi-dentate ligand and a three-dimensional structure was recently obtained (Liu 2008) of the compound formed by the reaction of this ligand with AgSbF_6_. The title one-dimensional chain structure (I) was formed when the ligand was reacted with silver(I)trifluoromethanesulfonate. It appears that the counter anions of Ag(I) complexes play a key role in the type of crystal structures formed with reactions of 1,2-bis[(3-cyanobenzylidene)hydrazono]-1,2-diphenylethane as a ligand. Herein the title crystal structure is reported.

Fig. 1 and Table 1 reveal that the coordination geometry for Ag1 and Ag2 is a 'T-shape' formed by two N atoms and a O atom, in which the two N atoms come from cyano and hydrazo groups, respectively, and the O atom is from trifluoromethanesulfonate ligand. In the crystal structure each 1,2-bis[(3-cyanobenzylidene)hydrazono]-1,2-diphenylethane ligand functions as tetradentate bridging ligand and coordinates to four Ag^I^ cations, which leads to the formation of a one-dimensional chain structure as shown in Fig. 2. In this one-dimensional chain there is 24-membered ring that includes two Ag^I^ ions. In addition, there are weak C—H···O non-classic hydrogen bonds in the chain and between neighboring chains (see Fig. 3 and Table 2).

### Experimental

8 ml benzene solution of AgSO_3_CF_3_ (0.0128 g, 0.05 mmol) was added very slowly on the 8 ml dichloromethane solution of 1,2-bis[(3-cyanobenzylidene)hydrazono]-1,2-diphenylethane (0.0093 g, 0.02 mmol). Colorless single crystals were obtained after the solution had been allowed to stand at room temperature for one month.

### Refinement

The H atoms were placed in calculated positions with C—H = 0.93 Å, and refined as riding with *U*_iso_(H) = 1.2*U*_eq_(C).

### Crystal data

**Table:**

[Ag_2_(CF_3_O_3_S)_2_(C_30_H_20_N_6_)]·C_6_H_6_	*F*_000_ = 2096
*M**_r_* = 1056.51	*D*_x_ = 1.716 Mg m^−^^3^
Monoclinic, *P*2_1_/*n*	Mo *K*α radiation λ = 0.71073 Å
Hall symbol: -P 2yn	Cell parameters from 7186 reflections
*a* = 10.8308 (9) Å	θ = 2.2–27.5º
*b* = 22.0864 (18) Å	µ = 1.14 mm^−^^1^
*c* = 17.0949 (13) Å	*T* = 298 (2) K
β = 90.420 (2)º	Block, colorless
*V* = 4089.2 (6) Å^3^	0.33 × 0.23 × 0.12 mm
*Z* = 4	

### Data collection

**Table:**

Bruker SMART APEX CCD diffractometer	8891 independent reflections
Radiation source: fine-focus sealed tube	6637 reflections with *I* > 2σ(*I*)
Monochromator: graphite	*R*_int_ = 0.026
*T* = 298(2) K	θ_max_ = 27.0º
φ and ω scans	θ_min_ = 2.1º
Absorption correction: multi-scan(SADABS; Sheldrick, 1996)	*h* = −13→13
*T*_min_ = 0.705, *T*_max_ = 0.875	*k* = −28→23
23919 measured reflections	*l* = −20→21

### Refinement

**Table:**

Refinement on *F*^2^	Secondary atom site location: difference Fourier map
Least-squares matrix: full	Hydrogen site location: inferred from neighbouring sites
*R*[*F*^2^ > 2σ(*F*^2^)] = 0.048	H-atom parameters constrained
*wR*(*F*^2^) = 0.134	*w* = 1/[σ^2^(*F*_o_^2^) + (0.0788*P*)^2^ + 0.28*P*] where *P* = (*F*_o_^2^ + 2*F*_c_^2^)/3
*S* = 1.04	(Δ/σ)_max_ = 0.002
8891 reflections	Δρ_max_ = 1.16 e Å^−^^3^
541 parameters	Δρ_min_ = −0.29 e Å^−^^3^
Primary atom site location: structure-invariant direct methods	Extinction correction: none

### Special details

**Table:**

Geometry. All e.s.d.'s (except the e.s.d. in the dihedral angle between two l.s. planes) are estimated using the full covariance matrix. The cell e.s.d.'s are taken into account individually in the estimation of e.s.d.'s in distances, angles and torsion angles; correlations between e.s.d.'s in cell parameters are only used when they are defined by crystal symmetry. An approximate (isotropic) treatment of cell e.s.d.'s is used for estimating e.s.d.'s involving l.s. planes.
Refinement. Refinement of *F*^2^ against ALL reflections. The weighted *R*-factor *wR* and goodness of fit *S* are based on *F*^2^, conventional *R*-factors *R* are based on *F*, with *F* set to zero for negative *F*^2^. The threshold expression of *F*^2^ > σ(*F*^2^) is used only for calculating *R*-factors(gt) *etc*. and is not relevant to the choice of reflections for refinement. *R*-factors based on *F*^2^ are statistically about twice as large as those based on *F*, and *R*- factors based on ALL data will be even larger.

### Fractional atomic coordinates and isotropic or equivalent isotropic displacement parameters (Å^2^)

**Table:**

	*x*	*y*	*z*	*U*_iso_*/*U*_eq_	
Ag1	0.60132 (2)	0.346047 (14)	0.842000 (17)	0.05493 (12)	
Ag2	−0.00966 (3)	0.327873 (17)	0.633415 (18)	0.06544 (13)	
C1	0.2343 (3)	0.2034 (2)	1.0453 (2)	0.0601 (10)	
C2	0.1395 (3)	0.24169 (17)	0.9271 (2)	0.0477 (8)	
H2	0.2094	0.2652	0.9189	0.057*	
C3	0.1336 (3)	0.20407 (18)	0.9907 (2)	0.0503 (9)	
C4	0.0299 (4)	0.16746 (19)	1.0033 (3)	0.0601 (10)	
H4	0.0275	0.1413	1.0458	0.072*	
C5	−0.0677 (4)	0.1707 (2)	0.9522 (3)	0.0641 (11)	
H5	−0.1374	0.1471	0.9603	0.077*	
C6	−0.0627 (3)	0.20939 (19)	0.8882 (2)	0.0542 (9)	
H6	−0.1297	0.2116	0.8540	0.065*	
C7	0.0405 (3)	0.24457 (16)	0.87478 (18)	0.0425 (7)	
C8	0.1699 (5)	0.1599 (3)	0.7178 (4)	0.0954 (18)	
H8	0.2471	0.1772	0.7265	0.115*	
C9	0.0980 (6)	0.1804 (3)	0.6561 (4)	0.0965 (17)	
H9	0.1288	0.2100	0.6229	0.116*	
C10	−0.0162 (6)	0.1579 (3)	0.6435 (4)	0.0970 (18)	
H10	−0.0650	0.1726	0.6027	0.116*	
C11	−0.0582 (6)	0.1141 (4)	0.6903 (4)	0.112 (2)	
H11	−0.1370	0.0986	0.6817	0.135*	
C12	0.0122 (5)	0.0917 (3)	0.7507 (4)	0.0990 (17)	
H12	−0.0180	0.0607	0.7820	0.119*	
C13	0.1311 (5)	0.1160 (3)	0.7651 (3)	0.0882 (15)	
H13	0.1803	0.1018	0.8060	0.106*	
C14	0.3169 (4)	0.15481 (17)	0.4787 (2)	0.0525 (9)	
C15	0.4171 (3)	0.16303 (16)	0.5341 (2)	0.0470 (8)	
C16	0.5166 (4)	0.12303 (19)	0.5335 (2)	0.0605 (10)	
H16	0.5211	0.0927	0.4959	0.073*	
C17	0.4111 (3)	0.20912 (16)	0.58766 (18)	0.0410 (7)	
H17	0.3453	0.2362	0.5865	0.049*	
C18	0.6075 (4)	0.1290 (2)	0.5888 (3)	0.0736 (13)	
H18	0.6734	0.1020	0.5895	0.088*	
C19	0.6024 (3)	0.1745 (2)	0.6435 (2)	0.0616 (11)	
H19	0.6651	0.1782	0.6806	0.074*	
C20	0.0456 (3)	0.28326 (16)	0.80567 (19)	0.0455 (8)	
H20	−0.0220	0.2854	0.7719	0.055*	
C21	0.5039 (3)	0.21506 (17)	0.64378 (19)	0.0450 (8)	
C22	0.5014 (3)	0.26226 (17)	0.70353 (19)	0.0459 (8)	
H22	0.5642	0.2635	0.7409	0.055*	
C23	−0.0077 (6)	0.3590 (3)	0.3969 (3)	0.0921 (16)	
C24	0.2354 (3)	0.38032 (15)	0.71347 (18)	0.0381 (7)	
C25	0.2541 (3)	0.42213 (16)	0.64852 (18)	0.0413 (7)	
C26	0.1591 (3)	0.44182 (19)	0.5994 (2)	0.0589 (10)	
H26	0.0792	0.4278	0.6074	0.071*	
C27	0.1816 (4)	0.4814 (2)	0.5398 (3)	0.0678 (12)	
H27	0.1170	0.4927	0.5067	0.081*	
C28	0.2958 (4)	0.5046 (2)	0.5275 (2)	0.0652 (11)	
H28	0.3097	0.5318	0.4869	0.078*	
C29	0.3915 (4)	0.4868 (2)	0.5771 (2)	0.0635 (11)	
H29	0.4701	0.5028	0.5704	0.076*	
C30	0.3710 (3)	0.44609 (18)	0.6354 (2)	0.0507 (9)	
H30	0.4365	0.4340	0.6672	0.061*	
C31	0.3343 (3)	0.37729 (15)	0.77589 (17)	0.0375 (7)	
C32	0.3266 (3)	0.41966 (16)	0.84254 (19)	0.0425 (7)	
C33	0.3737 (3)	0.40375 (19)	0.9156 (2)	0.0543 (9)	
H33	0.4082	0.3656	0.9230	0.065*	
C34	0.3697 (4)	0.4441 (2)	0.9768 (2)	0.0688 (12)	
H34	0.4035	0.4334	1.0251	0.083*	
C35	0.2693 (4)	0.4756 (2)	0.8338 (2)	0.0682 (11)	
H35	0.2333	0.4863	0.7862	0.082*	
C36	0.2660 (6)	0.5154 (2)	0.8967 (3)	0.0917 (16)	
H36	0.2291	0.5532	0.8907	0.110*	
C37	0.3166 (5)	0.4996 (2)	0.9673 (3)	0.0820 (14)	
H37	0.3147	0.5268	1.0089	0.098*	
C38	0.8438 (8)	0.4793 (3)	0.7793 (4)	0.113 (2)	
F1	0.9530 (6)	0.4644 (4)	0.7917 (5)	0.262 (4)	
F2	0.7781 (7)	0.4824 (2)	0.8418 (3)	0.209 (3)	
F3	−0.0577 (5)	0.3522 (2)	0.3282 (3)	0.1588 (19)	
F4	0.0751 (5)	0.40079 (18)	0.3889 (2)	0.1527 (17)	
F5	−0.0909 (5)	0.37934 (18)	0.4451 (3)	0.1540 (17)	
F6	0.8393 (6)	0.5347 (2)	0.7509 (3)	0.185 (2)	
N1	0.1370 (2)	0.34714 (13)	0.72112 (16)	0.0422 (6)	
N2	0.1421 (2)	0.31398 (13)	0.79151 (16)	0.0432 (6)	
N3	0.3146 (3)	0.2046 (2)	1.0884 (2)	0.0801 (12)	
N4	0.2379 (3)	0.14639 (16)	0.4371 (2)	0.0646 (9)	
N5	0.4273 (2)	0.34166 (13)	0.76968 (15)	0.0399 (6)	
N6	0.4161 (2)	0.30172 (13)	0.70622 (15)	0.0435 (6)	
O1	0.7827 (4)	0.37030 (17)	0.7513 (2)	0.0958 (12)	
O2	0.6537 (4)	0.4471 (3)	0.6981 (4)	0.192 (3)	
O3	0.8540 (3)	0.43076 (19)	0.6441 (2)	0.0966 (11)	
O4	0.1109 (3)	0.30513 (16)	0.50631 (17)	0.0794 (9)	
O5	0.1467 (2)	0.27367 (15)	0.37305 (17)	0.0741 (9)	
O6	−0.0434 (3)	0.24803 (15)	0.4356 (2)	0.0845 (10)	
S1	0.77205 (10)	0.42631 (6)	0.70898 (8)	0.0746 (3)	
S2	0.05837 (9)	0.28870 (5)	0.43195 (6)	0.0573 (3)	

### Atomic displacement parameters (Å^2^)

**Table:**

	*U*^11^	*U*^22^	*U*^33^	*U*^12^	*U*^13^	*U*^23^
Ag1	0.04308 (17)	0.0677 (2)	0.05368 (19)	−0.00110 (12)	−0.02067 (13)	−0.00059 (13)
Ag2	0.0565 (2)	0.0816 (3)	0.0578 (2)	−0.01519 (15)	−0.02769 (15)	0.00643 (15)
C1	0.044 (2)	0.084 (3)	0.052 (2)	0.0114 (19)	0.0044 (18)	0.019 (2)
C2	0.0314 (16)	0.060 (2)	0.0514 (19)	−0.0020 (15)	−0.0010 (14)	0.0036 (17)
C3	0.0407 (18)	0.062 (2)	0.0479 (19)	0.0072 (16)	−0.0016 (15)	0.0075 (17)
C4	0.061 (2)	0.062 (3)	0.058 (2)	0.0010 (19)	0.012 (2)	0.0121 (19)
C5	0.044 (2)	0.076 (3)	0.072 (3)	−0.0169 (19)	0.009 (2)	0.004 (2)
C6	0.0375 (18)	0.073 (3)	0.052 (2)	−0.0085 (17)	−0.0026 (15)	−0.0030 (19)
C7	0.0337 (15)	0.053 (2)	0.0404 (17)	−0.0005 (14)	−0.0043 (13)	−0.0016 (14)
C8	0.066 (3)	0.109 (5)	0.112 (5)	−0.009 (3)	0.011 (3)	−0.025 (4)
C9	0.091 (4)	0.080 (4)	0.118 (5)	0.006 (3)	0.022 (4)	−0.003 (3)
C10	0.102 (4)	0.085 (4)	0.103 (4)	0.005 (3)	−0.030 (4)	0.005 (3)
C11	0.083 (4)	0.126 (6)	0.128 (5)	−0.018 (4)	−0.025 (4)	−0.008 (5)
C12	0.095 (4)	0.090 (4)	0.112 (4)	−0.009 (3)	0.012 (4)	0.001 (3)
C13	0.093 (4)	0.095 (4)	0.077 (3)	0.004 (3)	−0.007 (3)	−0.012 (3)
C14	0.054 (2)	0.055 (2)	0.048 (2)	0.0003 (17)	−0.0112 (18)	−0.0032 (16)
C15	0.0454 (18)	0.053 (2)	0.0421 (18)	0.0015 (15)	−0.0101 (15)	0.0029 (15)
C16	0.062 (2)	0.063 (3)	0.056 (2)	0.016 (2)	−0.0106 (19)	−0.0164 (19)
C17	0.0342 (15)	0.050 (2)	0.0381 (16)	0.0067 (14)	−0.0057 (13)	−0.0013 (14)
C18	0.061 (3)	0.077 (3)	0.082 (3)	0.027 (2)	−0.020 (2)	−0.024 (2)
C19	0.047 (2)	0.078 (3)	0.060 (2)	0.0223 (19)	−0.0180 (18)	−0.011 (2)
C20	0.0355 (16)	0.057 (2)	0.0440 (18)	−0.0007 (15)	−0.0102 (14)	−0.0013 (16)
C21	0.0361 (16)	0.057 (2)	0.0417 (18)	0.0055 (14)	−0.0062 (14)	−0.0022 (15)
C22	0.0358 (16)	0.062 (2)	0.0402 (17)	0.0055 (15)	−0.0108 (14)	−0.0021 (15)
C23	0.115 (5)	0.083 (4)	0.078 (4)	0.020 (3)	0.000 (3)	0.006 (3)
C24	0.0321 (15)	0.0444 (19)	0.0378 (16)	0.0053 (13)	−0.0030 (13)	−0.0060 (14)
C25	0.0374 (16)	0.049 (2)	0.0373 (16)	0.0038 (14)	−0.0048 (13)	−0.0029 (14)
C26	0.0455 (19)	0.063 (3)	0.068 (2)	−0.0023 (17)	−0.0177 (18)	0.014 (2)
C27	0.061 (2)	0.068 (3)	0.073 (3)	0.002 (2)	−0.024 (2)	0.026 (2)
C28	0.072 (3)	0.069 (3)	0.055 (2)	0.004 (2)	0.002 (2)	0.015 (2)
C29	0.051 (2)	0.089 (3)	0.051 (2)	−0.007 (2)	0.0028 (17)	0.015 (2)
C30	0.0399 (17)	0.074 (3)	0.0385 (18)	0.0030 (17)	−0.0010 (14)	0.0049 (17)
C31	0.0326 (15)	0.0447 (19)	0.0351 (16)	−0.0020 (13)	−0.0044 (12)	0.0012 (13)
C32	0.0385 (16)	0.048 (2)	0.0413 (17)	−0.0054 (14)	−0.0021 (14)	−0.0046 (14)
C33	0.0493 (19)	0.068 (3)	0.046 (2)	−0.0020 (18)	−0.0029 (16)	−0.0052 (18)
C34	0.077 (3)	0.086 (3)	0.044 (2)	−0.008 (2)	−0.007 (2)	−0.009 (2)
C35	0.093 (3)	0.063 (3)	0.048 (2)	0.018 (2)	−0.004 (2)	−0.0052 (19)
C36	0.145 (5)	0.061 (3)	0.069 (3)	0.021 (3)	0.009 (3)	−0.011 (2)
C37	0.123 (4)	0.073 (3)	0.050 (2)	−0.008 (3)	0.010 (3)	−0.018 (2)
C38	0.129 (6)	0.105 (5)	0.104 (5)	−0.002 (4)	0.017 (4)	−0.011 (4)
F1	0.155 (4)	0.333 (10)	0.295 (8)	0.017 (5)	−0.108 (5)	−0.147 (7)
F2	0.396 (8)	0.116 (3)	0.116 (3)	−0.016 (4)	0.116 (4)	−0.022 (3)
F3	0.196 (4)	0.167 (4)	0.112 (3)	0.078 (3)	−0.057 (3)	−0.001 (3)
F4	0.237 (5)	0.088 (3)	0.133 (3)	−0.040 (3)	0.015 (3)	0.030 (2)
F5	0.208 (4)	0.099 (3)	0.156 (3)	0.081 (3)	0.068 (3)	0.012 (2)
F6	0.296 (7)	0.100 (3)	0.159 (4)	−0.057 (4)	0.074 (4)	−0.009 (3)
N1	0.0327 (13)	0.0523 (17)	0.0416 (15)	0.0013 (12)	−0.0096 (12)	−0.0005 (12)
N2	0.0356 (14)	0.0517 (17)	0.0422 (15)	−0.0060 (12)	−0.0108 (12)	0.0046 (12)
N3	0.059 (2)	0.122 (4)	0.058 (2)	0.015 (2)	−0.0092 (18)	0.024 (2)
N4	0.064 (2)	0.071 (2)	0.059 (2)	0.0026 (17)	−0.0234 (18)	−0.0104 (17)
N5	0.0328 (13)	0.0513 (17)	0.0354 (14)	−0.0010 (11)	−0.0074 (11)	−0.0039 (12)
N6	0.0392 (14)	0.0516 (17)	0.0396 (15)	0.0040 (12)	−0.0085 (12)	−0.0077 (12)
O1	0.127 (3)	0.083 (2)	0.077 (2)	0.008 (2)	0.032 (2)	0.0112 (19)
O2	0.050 (2)	0.210 (6)	0.316 (9)	0.021 (3)	−0.007 (3)	0.021 (6)
O3	0.092 (2)	0.123 (3)	0.076 (2)	−0.005 (2)	0.0248 (19)	0.014 (2)
O4	0.081 (2)	0.096 (2)	0.0608 (18)	−0.0149 (18)	−0.0072 (15)	−0.0070 (16)
O5	0.0473 (14)	0.105 (2)	0.0699 (18)	0.0017 (15)	−0.0006 (14)	−0.0231 (17)
O6	0.0638 (18)	0.078 (2)	0.112 (3)	−0.0142 (16)	0.0123 (18)	−0.0275 (19)
S1	0.0487 (6)	0.0939 (9)	0.0814 (8)	0.0003 (5)	0.0038 (5)	0.0091 (7)
S2	0.0507 (5)	0.0619 (6)	0.0591 (6)	−0.0009 (4)	−0.0021 (4)	−0.0120 (5)

### Geometric parameters (Å, °)

**Table:**

Ag1—N4^i^	2.196 (3)	C22—H22	0.9300
Ag1—N5	2.249 (3)	C23—F4	1.295 (7)
Ag1—O1	2.568 (3)	C23—F3	1.299 (7)
Ag2—N3^ii^	2.169 (3)	C23—F5	1.305 (6)
Ag2—N1	2.217 (3)	C23—S2	1.810 (6)
Ag2—O4	2.592 (3)	C24—N1	1.300 (4)
C1—N3	1.137 (5)	C24—C25	1.459 (5)
C1—C3	1.430 (5)	C24—C31	1.508 (4)
C2—C3	1.371 (5)	C25—C30	1.392 (5)
C2—C7	1.393 (4)	C25—C26	1.392 (4)
C2—H2	0.9300	C26—C27	1.366 (6)
C3—C4	1.401 (5)	C26—H26	0.9300
C4—C5	1.368 (6)	C27—C28	1.356 (6)
C4—H4	0.9300	C27—H27	0.9300
C5—C6	1.388 (6)	C28—C29	1.391 (6)
C5—H5	0.9300	C28—H28	0.9300
C6—C7	1.382 (5)	C29—C30	1.363 (5)
C6—H6	0.9300	C29—H29	0.9300
C7—C20	1.459 (5)	C30—H30	0.9300
C8—C13	1.334 (8)	C31—N5	1.283 (4)
C8—C9	1.381 (9)	C31—C32	1.477 (4)
C8—H8	0.9300	C32—C35	1.390 (5)
C9—C10	1.349 (8)	C32—C33	1.390 (5)
C9—H9	0.9300	C33—C34	1.376 (5)
C10—C11	1.337 (9)	C33—H33	0.9300
C10—H10	0.9300	C34—C37	1.364 (7)
C11—C12	1.372 (8)	C34—H34	0.9300
C11—H11	0.9300	C35—C36	1.390 (6)
C12—C13	1.415 (8)	C35—H35	0.9300
C12—H12	0.9300	C36—C37	1.368 (7)
C13—H13	0.9300	C36—H36	0.9300
C14—N4	1.124 (5)	C37—H37	0.9300
C14—C15	1.447 (5)	C38—F1	1.244 (8)
C15—C17	1.371 (5)	C38—F2	1.290 (7)
C15—C16	1.394 (5)	C38—F6	1.317 (8)
C16—C18	1.366 (5)	C38—S1	1.846 (8)
C16—H16	0.9300	N1—N2	1.410 (4)
C17—C21	1.391 (4)	N3—Ag2^i^	2.169 (3)
C17—H17	0.9300	N4—Ag1^ii^	2.196 (3)
C18—C19	1.374 (6)	N5—N6	1.403 (4)
C18—H18	0.9300	O1—S1	1.437 (4)
C19—C21	1.393 (5)	O2—S1	1.373 (4)
C19—H19	0.9300	O3—S1	1.429 (3)
C20—N2	1.271 (4)	O4—S2	1.435 (3)
C20—H20	0.9300	O5—S2	1.433 (3)
C21—C22	1.460 (5)	O6—S2	1.423 (3)
C22—N6	1.271 (4)		
			
N4^i^—Ag1—N5	165.35 (12)	F5—C23—S2	111.1 (4)
N4^i^—Ag1—O1	85.26 (13)	N1—C24—C25	123.5 (3)
N5—Ag1—O1	108.59 (11)	N1—C24—C31	118.8 (3)
N3^ii^—Ag2—N1	157.73 (13)	C25—C24—C31	117.7 (3)
N3^ii^—Ag2—O4	94.85 (11)	C30—C25—C26	116.9 (3)
N1—Ag2—O4	103.97 (10)	C30—C25—C24	119.6 (3)
N3—C1—C3	178.0 (5)	C26—C25—C24	123.4 (3)
C3—C2—C7	119.8 (3)	C27—C26—C25	121.0 (4)
C3—C2—H2	120.1	C27—C26—H26	119.5
C7—C2—H2	120.1	C25—C26—H26	119.5
C2—C3—C4	120.9 (3)	C28—C27—C26	121.7 (4)
C2—C3—C1	119.0 (3)	C28—C27—H27	119.2
C4—C3—C1	120.1 (4)	C26—C27—H27	119.2
C5—C4—C3	119.2 (4)	C27—C28—C29	118.3 (4)
C5—C4—H4	120.4	C27—C28—H28	120.8
C3—C4—H4	120.4	C29—C28—H28	120.8
C4—C5—C6	120.1 (4)	C30—C29—C28	120.5 (4)
C4—C5—H5	120.0	C30—C29—H29	119.7
C6—C5—H5	120.0	C28—C29—H29	119.7
C7—C6—C5	120.9 (3)	C29—C30—C25	121.5 (3)
C7—C6—H6	119.6	C29—C30—H30	119.3
C5—C6—H6	119.6	C25—C30—H30	119.3
C6—C7—C2	119.2 (3)	N5—C31—C32	120.1 (3)
C6—C7—C20	120.0 (3)	N5—C31—C24	121.5 (3)
C2—C7—C20	120.9 (3)	C32—C31—C24	118.3 (3)
C13—C8—C9	121.5 (6)	C35—C32—C33	118.8 (3)
C13—C8—H8	119.2	C35—C32—C31	120.5 (3)
C9—C8—H8	119.2	C33—C32—C31	120.7 (3)
C10—C9—C8	120.8 (6)	C34—C33—C32	120.4 (4)
C10—C9—H9	119.6	C34—C33—H33	119.8
C8—C9—H9	119.6	C32—C33—H33	119.8
C11—C10—C9	119.1 (6)	C37—C34—C33	120.5 (4)
C11—C10—H10	120.4	C37—C34—H34	119.8
C9—C10—H10	120.4	C33—C34—H34	119.8
C10—C11—C12	121.4 (6)	C32—C35—C36	119.6 (4)
C10—C11—H11	119.3	C32—C35—H35	120.2
C12—C11—H11	119.3	C36—C35—H35	120.2
C11—C12—C13	119.6 (6)	C37—C36—C35	120.6 (5)
C11—C12—H12	120.2	C37—C36—H36	119.7
C13—C12—H12	120.2	C35—C36—H36	119.7
C8—C13—C12	117.4 (6)	C34—C37—C36	120.0 (4)
C8—C13—H13	121.3	C34—C37—H37	120.0
C12—C13—H13	121.3	C36—C37—H37	120.0
N4—C14—C15	177.3 (4)	F1—C38—F2	113.8 (8)
C17—C15—C16	121.1 (3)	F1—C38—F6	110.0 (8)
C17—C15—C14	119.4 (3)	F2—C38—F6	103.7 (6)
C16—C15—C14	119.5 (3)	F1—C38—S1	109.7 (5)
C18—C16—C15	119.1 (4)	F2—C38—S1	109.9 (6)
C18—C16—H16	120.4	F6—C38—S1	109.5 (6)
C15—C16—H16	120.4	C24—N1—N2	110.6 (3)
C15—C17—C21	119.5 (3)	C24—N1—Ag2	128.6 (2)
C15—C17—H17	120.3	N2—N1—Ag2	120.05 (19)
C21—C17—H17	120.3	C20—N2—N1	114.4 (3)
C16—C18—C19	120.6 (4)	C1—N3—Ag2^i^	152.6 (4)
C16—C18—H18	119.7	C14—N4—Ag1^ii^	164.0 (4)
C19—C18—H18	119.7	C31—N5—N6	112.7 (2)
C18—C19—C21	120.5 (3)	C31—N5—Ag1	125.7 (2)
C18—C19—H19	119.8	N6—N5—Ag1	121.30 (19)
C21—C19—H19	119.8	C22—N6—N5	113.6 (3)
N2—C20—C7	120.2 (3)	S1—O1—Ag1	115.1 (2)
N2—C20—H20	119.9	S2—O4—Ag2	126.35 (17)
C7—C20—H20	119.9	O2—S1—O3	117.2 (4)
C17—C21—C19	119.1 (3)	O2—S1—O1	115.3 (3)
C17—C21—C22	122.2 (3)	O3—S1—O1	113.7 (2)
C19—C21—C22	118.7 (3)	O2—S1—C38	105.4 (4)
N6—C22—C21	122.2 (3)	O3—S1—C38	101.6 (3)
N6—C22—H22	118.9	O1—S1—C38	100.8 (3)
C21—C22—H22	118.9	O6—S2—O5	113.91 (19)
F4—C23—F3	105.6 (5)	O6—S2—O4	115.0 (2)
F4—C23—F5	107.7 (6)	O5—S2—O4	114.75 (17)
F3—C23—F5	109.0 (6)	O6—S2—C23	104.5 (3)
F4—C23—S2	112.0 (5)	O5—S2—C23	103.3 (2)
F3—C23—S2	111.2 (4)	O4—S2—C23	103.3 (3)
			
C7—C2—C3—C4	1.2 (6)	C31—C32—C35—C36	−178.3 (4)
C7—C2—C3—C1	−177.7 (3)	C32—C35—C36—C37	−1.3 (8)
C2—C3—C4—C5	−1.7 (6)	C33—C34—C37—C36	0.4 (8)
C1—C3—C4—C5	177.2 (4)	C35—C36—C37—C34	−0.6 (9)
C3—C4—C5—C6	0.9 (6)	C25—C24—N1—N2	−176.7 (3)
C4—C5—C6—C7	0.5 (6)	C31—C24—N1—N2	1.3 (4)
C5—C6—C7—C2	−1.1 (6)	C25—C24—N1—Ag2	13.6 (5)
C5—C6—C7—C20	177.8 (4)	C31—C24—N1—Ag2	−168.4 (2)
C3—C2—C7—C6	0.2 (5)	N3^ii^—Ag2—N1—C24	−168.9 (4)
C3—C2—C7—C20	−178.6 (3)	O4—Ag2—N1—C24	44.2 (3)
C13—C8—C9—C10	2.4 (9)	N3^ii^—Ag2—N1—N2	22.2 (5)
C8—C9—C10—C11	−1.7 (10)	O4—Ag2—N1—N2	−124.6 (2)
C9—C10—C11—C12	−0.1 (11)	C7—C20—N2—N1	177.2 (3)
C10—C11—C12—C13	1.3 (10)	C24—N1—N2—C20	175.8 (3)
C9—C8—C13—C12	−1.1 (9)	Ag2—N1—N2—C20	−13.5 (4)
C11—C12—C13—C8	−0.7 (9)	C32—C31—N5—N6	176.0 (3)
C17—C15—C16—C18	2.0 (6)	C24—C31—N5—N6	−8.3 (4)
C14—C15—C16—C18	−176.3 (4)	C32—C31—N5—Ag1	−10.2 (4)
C16—C15—C17—C21	−1.9 (5)	C24—C31—N5—Ag1	165.5 (2)
C14—C15—C17—C21	176.4 (3)	N4^i^—Ag1—N5—C31	44.1 (6)
C15—C16—C18—C19	−1.3 (7)	O1—Ag1—N5—C31	−116.2 (3)
C16—C18—C19—C21	0.5 (8)	N4^i^—Ag1—N5—N6	−142.5 (4)
C6—C7—C20—N2	−177.4 (3)	O1—Ag1—N5—N6	57.1 (3)
C2—C7—C20—N2	1.5 (5)	C21—C22—N6—N5	177.6 (3)
C15—C17—C21—C19	1.0 (5)	C31—N5—N6—C22	−173.6 (3)
C15—C17—C21—C22	−178.5 (3)	Ag1—N5—N6—C22	12.3 (4)
C18—C19—C21—C17	−0.3 (6)	N4^i^—Ag1—O1—S1	−115.8 (2)
C18—C19—C21—C22	179.2 (4)	N5—Ag1—O1—S1	59.3 (3)
C17—C21—C22—N6	−2.2 (6)	N3^ii^—Ag2—O4—S2	10.7 (3)
C19—C21—C22—N6	178.3 (4)	N1—Ag2—O4—S2	178.7 (2)
N1—C24—C25—C30	−166.4 (3)	Ag1—O1—S1—O2	−24.1 (4)
C31—C24—C25—C30	15.6 (5)	Ag1—O1—S1—O3	−163.4 (2)
N1—C24—C25—C26	16.2 (5)	Ag1—O1—S1—C38	88.7 (3)
C31—C24—C25—C26	−161.8 (3)	F1—C38—S1—O2	−178.9 (7)
C30—C25—C26—C27	2.0 (6)	F2—C38—S1—O2	55.3 (7)
C24—C25—C26—C27	179.5 (4)	F6—C38—S1—O2	−58.0 (6)
C25—C26—C27—C28	−2.3 (7)	F1—C38—S1—O3	−56.2 (7)
C26—C27—C28—C29	0.5 (7)	F2—C38—S1—O3	178.0 (6)
C27—C28—C29—C30	1.3 (7)	F6—C38—S1—O3	64.6 (6)
C28—C29—C30—C25	−1.5 (7)	F1—C38—S1—O1	60.9 (7)
C26—C25—C30—C29	−0.2 (6)	F2—C38—S1—O1	−64.9 (6)
C24—C25—C30—C29	−177.8 (4)	F6—C38—S1—O1	−178.2 (5)
N1—C24—C31—N5	94.2 (4)	Ag2—O4—S2—O6	−40.0 (3)
C25—C24—C31—N5	−87.6 (4)	Ag2—O4—S2—O5	−175.0 (2)
N1—C24—C31—C32	−90.0 (4)	Ag2—O4—S2—C23	73.3 (3)
C25—C24—C31—C32	88.2 (4)	F4—C23—S2—O6	−178.6 (4)
N5—C31—C32—C35	147.9 (4)	F3—C23—S2—O6	−60.7 (5)
C24—C31—C32—C35	−27.9 (5)	F5—C23—S2—O6	60.9 (5)
N5—C31—C32—C33	−33.7 (5)	F4—C23—S2—O5	−59.2 (5)
C24—C31—C32—C33	150.5 (3)	F3—C23—S2—O5	58.8 (5)
C35—C32—C33—C34	−3.6 (6)	F5—C23—S2—O5	−179.6 (5)
C31—C32—C33—C34	178.0 (3)	F4—C23—S2—O4	60.7 (5)
C32—C33—C34—C37	1.7 (6)	F3—C23—S2—O4	178.6 (5)
C33—C32—C35—C36	3.3 (6)	F5—C23—S2—O4	−59.7 (5)

Symmetry codes: (i) *x*+1/2, −*y*+1/2, *z*+1/2; (ii) *x*−1/2, −*y*+1/2, *z*−1/2.

### Hydrogen-bond geometry (Å, °)

**Table:**

*D*—H···*A*	*D*—H	H···*A*	*D*···*A*	*D*—H···*A*
C33—H33···O6^i^	0.93	2.57	3.486 (5)	168
C26—H26···O3^iii^	0.93	2.52	3.406 (5)	159
C22—H22···O5^i^	0.93	2.56	3.382 (4)	148
C6—H6···O5^iv^	0.93	2.47	3.178 (4)	133

Symmetry codes: (i) *x*+1/2, −*y*+1/2, *z*+1/2; (iii) *x*−1, *y*, *z*; (iv) *x*−1/2, −*y*+1/2, *z*+1/2.
